# Self-assembled hybrid metal oxide base catalysts prepared by simply mixing with organic modifiers

**DOI:** 10.1038/ncomms9580

**Published:** 2015-10-05

**Authors:** Masazumi Tamura, Ryota Kishi, Yoshinao Nakagawa, Keiichi Tomishige

**Affiliations:** 1 Graduate School of Engineering, Tohoku University, Aoba 6-6-07, Aramaki, Aoba-ku, Sendai 980-8579, Japan

## Abstract

Multidentate materials formed by simply mixing heterogeneous and homogeneous components are promising for construction of versatile active sites on the surface of heterogeneous compounds, however, to the best of our knowledge, there are no reports on such materials. Self-assembly of hetero-hybrid catalytic materials occurs when heterogeneous catalysts having adjacent Lewis acid-Lewis base sites are mixed with an organic modifier that contains at least two Lewis base functional groups. Here we demonstrate the strategy by combining cerium oxide and 2-cyanopyridine that self-assembles to form a charge-transfer complex in methanol that exhibits a 2,000-fold increase in reaction rate for hydromethoxylation of acrylonitrile with high selectivity compared with cerium oxide or 2-cyanopyridine alone. The catalytic system is applied to the transesterification and Knoevenagel condensation affording 14-fold and 11-fold higher activity, respectively, than cerium oxide alone. These results demonstrate the potential versatility of the catalytic system and the generality of the catalyst preparation strategy.

## Abstract (web summary)

Self-assembled heterogeneous/homogeneous hybrid materials may offer ease of preparation and tunable catalytic performance. Here, the authors report the accelerated selective hydromethoxylation of acrylonitrile in the presence of CeO_2_ and 2-cyanopyridine, resulting from the proposed supramolecular complex.

## Graphical abstract

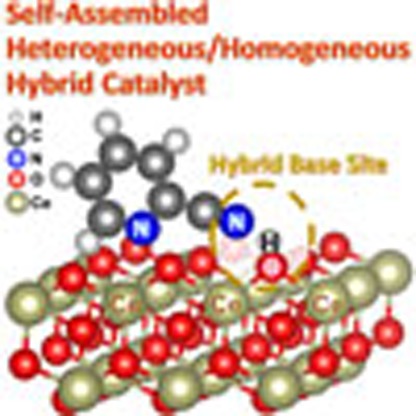

Catalysts are essential materials for developing efficient and environmental friendly processes in the chemical industry. The cooperation among two or more different materials is one of the most promising methods for preparing novel catalysts that are effective because the catalytic system has the potential to have enhanced properties that may not be observed in the individual materials^1^,^2^,^3^,^4^,^5^,^6^,^7^. Among various approaches, homogeneous–heterogeneous hybridization enables fine-tuning of the active site property at the molecular level by virtue of the flexibility and versatility of the organic components^4^,^5^,^6^,^7^,^8^,^9^,^10^,^11^,^12^. Immobilization of homogeneous catalysts on solid materials is a well-known and often-used technique^7^,^8^, however this approach requires cumbersome catalyst preparation procedures. In contrast, treatment of solid catalysts by addition of organic modifiers is an ideal method since it is simple and it has high versatility. Amines are widely used as organic modifiers for supported noble metal catalysts^13^, while heterogeneous Pd catalysts with quinoline derivatives are traditionally used as Lindlar or Rosenmund catalysts^14^. Baiker and co-workers have made comprehensive studies on the modification of noble metal catalysts with cinchona alkaloids for enantioselective hydrogenation^13^,^15^,^16^,^17^,^18^,^19^. Thiol has been applied as an organic modifier for noble metal nanoparticles, and thiol-modified catalysts are now well-known to form self-assembled monolayers^5^,^11^,^12^. Polymers^20^,^21^ and dendrimers^10^ are also reported to be effective modifiers for noble metals. On the other hand, there are few reports on the efficacious modification of metal oxide catalysts with an organic modifier^22^, although metal oxides are useful materials in many fields of chemistry owing to their acid–base and redox properties, as well as their high-thermal stability and durability. SiO_2_-TiO_2_ modified with *N*,*N*-dimethyl-butylamine exhibits a sixfold higher activity for epoxidation of allylic alcohols than SiO_2_-TiO_2_ alone, where *N*,*N*-dimethyl-butylamine has been suggested to work as a poison for the undesirable sites^22^. Therefore, design of an active site on a metal oxide surface by modification with organic modifiers could give new catalytic functions to metal oxides.

Polydentate compounds bearing multiple coordination sites have attracted much attention in the field of catalysis, sensing and biological chemistry^23^,^24^,^25^,^26^,^27^,^28^. Multidentate Lewis bases and multidentate Lewis acids are being actively studied^29^,^30^,^31^,^32^,^33^,^34^,^35^ because these multidentate Lewis bases or acids often enhance the Lewis basicity or acidity mainly by stabilization through the interaction between the spatially proximate active centres and the substrate, or by relief of the electronic or steric repulsion between active centres^28^. For instance, the basicity of 1,8-bis(dimethylamino)naphthalene is 10^7^-fold higher than that of aniline^26^, and the Lewis acidity of bidentate antimony Lewis acids is two orders of magnitude higher than mono antimony Lewis acid^32^. Therefore, assembly of multiple active centres can generate an effective active site even if the individual active sites are ineffective. Generally, these multidentate compounds are single complicated organic compounds with functional groups composed of 12–16 elements and they have low stability, are time-consuming to prepare and difficult to handle. If multidentate materials could be formed by simply mixing heterogeneous and homogeneous components such as solid materials and organic molecules, then new and versatile active sites could be formed on the surface of a heterogeneous compound, so that many of the above drawbacks would be overcome. However, there are no reports on multidentate materials composed of heterogeneous and homogeneous components.

In this article, we outline a strategy for design of organic compound-modified metal oxides. Our approach is based on the self-assembly of two Lewis base sites of a metal oxide and an organic modifier constructed on the surface of the metal oxide to form a hybrid base site (Fig. 1). Metal oxides have adjacent Lewis acid and Lewis base sites on the same surface, and therefore the Lewis acid site on the metal oxide surface can be used as an adsorption moiety for an organic modifier. If an organic molecule having weak and strong Lewis base functional groups is introduced onto the metal oxide, a new hybrid base site composed of two Lewis bases of an organic modifier and metal oxide surface might be formed through the proximity of these Lewis bases through interaction between the Lewis base functional group of the organic modifier and the Lewis acid site on the metal oxide surface. Recently, our group and other research groups have reported that CeO_2_ exhibits high catalytic activity in liquid-phase organic reactions at low temperatures (≤473 K), which is related to the unique acid–base property of CeO_2_ (refs 36, 37, 38, 39, 40, 41), particularly high basicity and moderate Lewis acidity^41^,^42^. We focus on the unique acid–base property of CeO_2_, and aim at constructing a heterogeneous/homogeneous hybrid catalyst through the interaction between Lewis acid sites on CeO_2_ and Lewis base functional groups of various organic compounds (Fig. 1b). Herein, we demonstrate that the catalyst complex composed of CeO_2_ and 2-cyanopyridine, readily self-assembles by simply mixing the compounds, and the resulting catalytic material exhibits dramatic activity rate enhancement for hydromethoxylation of acrylonitrile (reaction rate >2,000-fold compared with only CeO_2_ or 2-cyanopyridine).

## Results

### Catalyst screening in hydromethoxylation of acrylonitrile

As a first step toward developing the strategy, we investigated the combination of CeO_2_ and pyridine derivatives bearing various functional groups at the 2-position of the pyridine ring in hydromethoxylation of acrylonitrile to 3-methoxypropionitrile at 323 K (Table 1), which is known to be a typical addition reaction catalysed by a base and is useful for the synthesis of pharmaceutical intermediates, plasticizers and additives for synthetic rubber. Without pyridine derivatives, using only CeO_2_ provided low yield (0.5%) even after 24 h reaction time (Table 1, entry 13). Addition of pyridine gave slightly higher activity than that without pyridine derivatives (Table 1, entry 5), and the activity was similar to the activity of only pyridine (without CeO_2_) as a base catalyst (reaction rate of only pyridine: 0.04 mmol h^−1^ g^−1^). Pyridine derivatives with OH, CH_3_ or OCH_3_ groups showed two to fourfold higher activity than that in the case of pyridine (Table 1, entries 2–4). In contrast, pyridine derivatives with COCH_3_, CH_2_OH, C_2_H_5_, CONH_2_ or CH_2_OCH_3_ showed low activity (Table 1, entries 6–10). The pyridine derivative, 2-cyanopyridine, which has a cyano group at the 2-position of the pyridine ring, showed far higher activity with high selectivity for the product (>99%) than the other pyridine derivatives (Table 1, entry 1), and the activity was >2,000-fold higher than only CeO_2_ (Table 1, entries 1 and 13). Only 2-cyanopyridine (without CeO_2_) provided no product (<0.1%, 24 h). Therefore, the combination of CeO_2_ and 2-cyanopyridine was effective for the reaction, and the rate enhancement (>2,000-fold) is the highest among previous reports on heterogeneous and homogeneous hybrid catalysts (maximum rate enhancement, ∼100-fold with Pt/SiO_2_+cinchonidine^43^).

**Figure 2:**
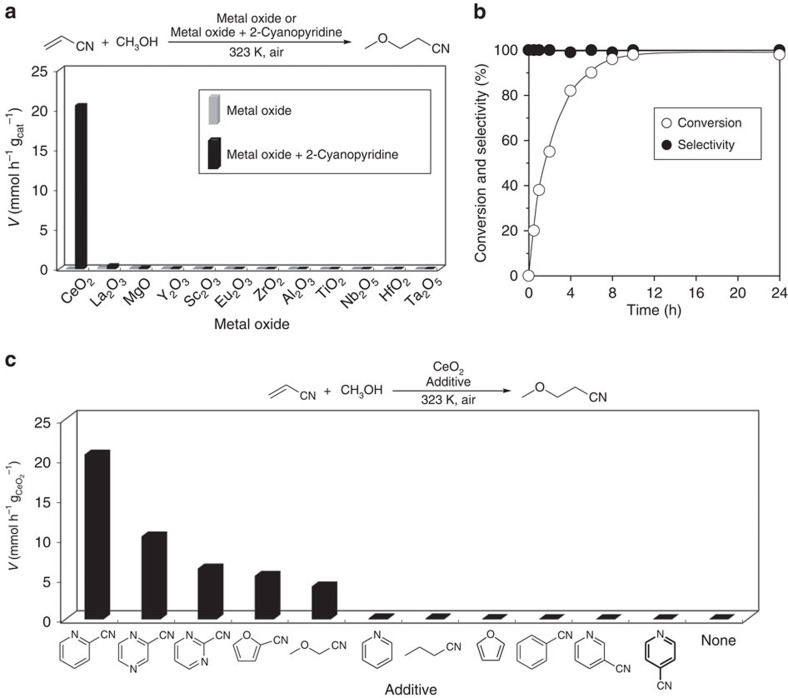
Metal oxides and nitriles screening and the time course. (**a**) Comparison of the combination of metal oxide catalysts and 2-cyanopyridine. Reaction conditions without 2-cyanopyridine (grey bar): acrylonitrile (10 mmol), methanol (20 mmol), metal oxide (172 mg), 323 K, air, 12–48 h. Reaction conditions with 2-cyanopyridine (black bar): acrylonitrile (10 mmol), methanol (20 mmol), metal oxide (172 mg), 2-cyanopyridine (2 mmol), 323 K, air, 0.5–48 h. Detailed data are described in Supplementary Tables 1 and 2. (**b**) Time course of the reaction over CeO_2_+2-cyanopyridine hybrid catalyst. Reaction conditions: acrylonitrile (10 mmol), methanol (15 mmol), CeO_2_ (1 mmol), 2-cyanopyridine (1 mmol), 323 K, air. Detailed data are described in Supplementary Table 3. (**c**), Effect of organic compounds on the activity. Reaction conditions: acrylonitrile (10 mmol), methanol (20 mmol), CeO_2_ (1 mmol), organic additive (2 mmol), 323 K, air. Detailed data are described in Supplementary Table 4.

To confirm the necessity of CeO_2_ in this system, various metal oxides were applied to the reaction. Figure 2a shows the reaction rates (*V*/mmol h^−1^ g_cat_^−1^) for cases of only metal oxides and the hybrid system of metal oxides+2-cyanopyridine. Detailed results are shown in the Supplementary Tables 1 and 2. Rate enhancement was hardly observed on the other metal oxides, although some rate enhancement was observed on La_2_O_3_ (about twofold), indicating that the combination of CeO_2_ and 2-cyanopyridine is essential to achieving high activity in this reaction system. The time course of the reaction over CeO_2_+2-cyanopyridine was investigated (Fig. 2b). Detailed results are shown in the Supplementary Table 3. The reaction proceeded smoothly to reach almost 100% conversion in 12 h with >99% selectivity, affording 98% yield of the product. High selectivity was maintained at high conversion for prolonged reaction time (24 h). To verify that the observed catalysis was not derived from Ce species leached into the solution, the reaction was conducted for 0.5 h to afford 17% conversion, followed by removal of CeO_2_ from the reaction mixture by hot filtration. The filtrate was reacted under the same conditions, and the reaction completely stopped (Supplementary Fig. 1). Inductive coupled plasma results confirmed that the Ce species in the solution was below the detection limit (<0.1 p.p.m.), which demonstrated that catalytically active species did not elute from CeO_2_ under the reaction conditions and the observed catalysis was truly heterogeneous. The number of Ce cations on CeO_2_ surface was calculated to be 1.13 mmol g^−1^ from the surface density of Ce atoms on CeO_2_ (111) and from its surface area (86 m^2^ g^−1^) (ref. 37). On the basis of the total amount of CeO_2_ and the number of Ce cations on CeO_2_ surface, the turnover numbers were calculated to be 9.8 and 50, respectively, so that CeO_2_+2-cyanopyridine catalytically promoted the reaction. Finally, the effect of nitrile compounds, pyridine and furan was examined in the same reaction (Fig. 2c). Detailed results are shown in the Supplementary Table 4. Heteroaromatic compounds bearing a cyano group at the 2-position of the heteroaromatic ring, 2-cyanopyridine, cyanopyrazine and 2-cyanopyrimidine, showed high activity with the order of the activity following, 2-cyanopyridine>cyanopyrazine>2-cyanopyrimidine. Addition of 2-furonitrile or methoxyacetonitrile was also effective for the catalytic system. On the other hand, additives without a cyano group, such as pyridine and furan, hardly provided the product, while additives without any other heteroatoms (N or O) except for the cyano group, butyronitrile and benzonitrile, provided almost no product, indicating that both a cyano group and a heteroatom (N or O) were essential constituents of additives that were effective. The structural isomers of 2-cyanopyridine, namely 3- and 4-cyanopyridine, were not effective in the system, suggesting that the relative position between the cyano group and the heteroatom (N or O) is of great importance. This means that the effective organic additives have common structural features; effective nitriles have a cyano group at the α-position of a heteroatom (N or O). To ascertain whether the basicity of the additives is related to the reaction rate (*V*), the basicity was estimated by DFT calculation as Δ*E* of protonation (Supplementary Table 5). No clear correlation between the basicity and the reaction rate was observed in all additives, and only the basicity cannot explain the rate enhancement effect of the additives, which shows that two base functional groups at the suitable position in the additive structure are necessary for effective nitrile additives. Focusing on the effective nitrile additives (2-cyanopyridine, cyanopyrazine, 5-fluoro-2-cyanopyridine, 2-cyanopyrimidine, 2-furonitrile and methoxylacetonitrile), moderate correlation between the reaction rate and the higher basicity among the base functional groups (lower Δ*E*) was observed. However, the effective nitriles can be divided into two types, nitriles with another N atom (2-cyanopyridine, cyanopyrazine, 5-fluoro-2-cyanopyridine and 2-cyanopyrimidine) and nitriles with an O atom (2-furonitrile and methoxylacetonitrile). In the case of the former nitriles (2-cyanopyridine, cyanopyrazine, 5-fluoro-2-cyanopyridine and 2-cyanopyrimidine), the basicity of the N atom in the heteroaromatic ring is higher than that of the N atom of the cyano group, in contrast, in the case of the latter nitriles (2-furonitrile and methoxylacetonitrile), the basicity of the O atom is lower than that of the N atom in the cyano group. In addition, focusing on the basicity of the cyano group in each type of effective nitriles, the higher basicity of the cyano group seems to be effective for the catalytic system (2-cyanopyridine versus cyanopyrazine, 5-fluoro-2-cyanopyridine or 2-cyanopyrimidine, 2-furonitrile versus methoxyaetonitrile). In particular, 5-fluoro-2-cyanopyridine, electron-withdrawing fluorine substituted 2-cyanopyridine at the 5-position in the pyridine ring, provided lower basicity of the cyano group than 2-cyanopyridine, and the reaction rate also decreased (Supplementary Table 5), which supports that the higher basicity of the cyano group is effective for the catalytic system. However, the effect of the basicity of the base functional groups in organic modifiers will be pretty complex, and systematic studies on the effect of nitrile additives including substituent effects or steric effects will be explored in the future to clarify the effect of basicity.

The reusability of CeO_2_ was investigated. CeO_2_ was easily retrieved from the reaction mixture by filtration. After washing with acetone, followed by calcining at 873 K for 3 h, the recovered CeO_2_ was used for the next reaction. CeO_2_ could be reused at least three times without remarkable loss of activity and selectivity (Supplementary Fig. 2), and x-ray diffraction (XRD) analyses confirmed that the structure of CeO_2_ remained intact during the reusability tests (Supplementary Fig. 3).

### Investigation of adsorption state of 2-cyanopyridine on CeO_2_

**Figure 3:**
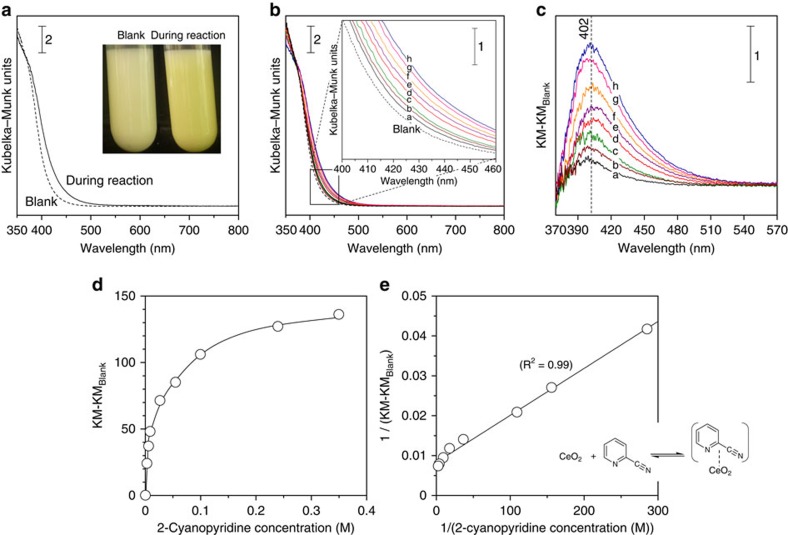
Analyses of the reaction mixtures with UV–vis spectroscopy. (**a**) UV–vis spectra of the reaction mixtures with and without 2-cyanopyridine. Reaction conditions: acrylonitrile (10 mmol), methanol (20 mmol), CeO_2_ (1 mmol), 2-cyanopyridine (blank: 0 mmol, reaction: 2 mmol), 323 K, air, 0.25 h. (**b**), UV–vis spectra of the mixtures at various 2-cyanopyridine concentrations. Conditions: methanol (20 mmol), CeO_2_ (1 mmol), 2-cyanopyridine (**a**: 0.0035 M; **b**: 0.0064 M; **c**: 0.0091 M; **d**: 0.027 M; **e**: 0.055 M; **f**: 0.10 M; **g**: 0.24 M; **h**: 0.35 M), 323 K, air, 1.0 h. (**c**), Difference spectra obtained by subtraction of blank spectra (KM_Blank_) from each spectrum (KM) of **b**. (**d**) Band intensity at 402 nm in the difference spectra (KM-KM_Blank_) as a function of 2-cyanopyridine concentration. (**e**) Correlation between 1/(2-cyanopyridine concentration) and 1/(KM-KM_Blank_). KM, Kubelka–Munk.

The colour of metal oxides such as CeO_2_ and TiO_2_ is largely influenced by the electronic state, and such a colour change is often observed for adsorption of organic compounds onto metal oxides^44^,^45^,^46^. In the present reaction, the colour of CeO_2_ in methanol solvent was yellowish (Blank, see the inset of Fig. 3a), while the colour of CeO_2_ in methanol solvent became deep yellow almost instantaneously when 2-cyanopyridine was added to the CeO_2_+methanol solution. The corresponding change of UV–vis spectra was observed to have a maximum absorption peak at 402 nm (Fig. 3a). When CeO_2_ was removed from the solution by filtration, the residual liquid solution was clear and colourless. The absorption intensity increased with increasing amounts of 2-cyanopyridine (Fig. 3b). The difference spectra, which are obtained by subtracting the blank spectrum, showed that the band intensity increased with increasing amounts of 2-cyanopyridine. Neither CeO_2_ nor 2-cyanopyridine has absorption peaks in this range (Fig. 3a and Supplementary Fig. 4). Therefore, the colour change is derived from strong interaction between CeO_2_ and 2-cyanopyridine, and can be assigned to the ligand-to-metal charge transfer (C-T) between CeO_2_ and 2-cyanopyridine according to the reported literature on C-T complex formation between CeO_2_ or TiO_2_ and phenolic compounds^45^,^46^. The maximum position of the absorption band was almost unchanged (Fig. 3c), which indicates that the same adsorption species were formed on CeO_2_ regardless of the amount of 2-cyanopyridine present. The interpretation of this phenomenon is that the surface species formed by the interaction between CeO_2_ and 2-cyanopyridine are uniform in the range from low-to-high concentration. No colour change was observed by using benzonitrile or pyridine instead of 2-cyanopyridine, although benzonitrile or pyridine can be adsorbed onto Lewis acid sites of CeO_2_ at the N atom of the cyano group or at the pyridine ring, respectively^42^. The 2-cyanopyridine was easily removed from CeO_2_ by washing with large amounts of methanol (determined by TG-DTA (Supplementary Fig. 5), which means that 2-cyanopyridine does not react with the surface of CeO_2_. These results are evidence that 2-cyanopyridine is adsorbed onto CeO_2_ at both the N atoms of the cyano group and the pyridine ring without transformation of the cyano group in 2-cyanopyridine. The band intensity at 402 nm is plotted as a function of 2-cyanopyridine concentration (Fig. 3d). The band intensity increased linearly with an increase in the 2-cyanopyridine concentration, and leveled off at high concentrations (≥0.24 M). Good linear correlation between 1/(2-cyanopyridine concentration) and 1/(KM-KM_Blank_) was obtained (Fig. 3e), indicating that the complexation between 2-cyanopyridine and CeO_2_ follows Langmuir isotherm kinetics. The CeO_2_ and 2-cyanopyridine are in equilibrium with CeO_2_-2-cyanopyridine C-T complex (see inset of Fig. 3e), resulting in the observed colour change of CeO_2_. The UV–vis spectra were measured by varying 2-cyanopyridine/methanol ratios from 1/15 to ∞ (no methanol) (Supplementary Fig. 6). Although the spectrum with the ratio of ∞ (no methanol) had a wide absorption band that was different from that under standard reaction conditions, the other spectra were almost the same (2-cyanopyridine/methanol ratio=0.067, 0.1, 0.2, 1 and 2). Thus, the amount and electronic state of the formed CeO_2_-2-cyanopyridine C-T complex were almost the same provided that methanol was present, meaning that CeO_2_ and 2-cyanopyridine self-assembled in the methanol solvent to form the same C-T complex.

To confirm the adsorption state of 2-cyanopyridine and methanol on CeO_2_, FTIR analyses were carried out by the introduction of methanol to CeO_2_, that was followed by the introduction of 2-cyanopyridine at 323 K (Supplementary Fig. 7). The bands at 2,235 and 2,287 cm^−1^ were observed in the region between 2,000 and 2,350 cm^−1^, which can be assigned to non-interactive *ν*(CN) and hydrogen-bonding *ν*(CN), respectively. Therefore, considering that the band at 2,287 cm^−1^ was not observed without methanol (Supplementary Fig. 7), introduction of methanol brings about hydrogen bond between the CN group in 2-cyanopyridine and the H atom.

**Figure 4:**
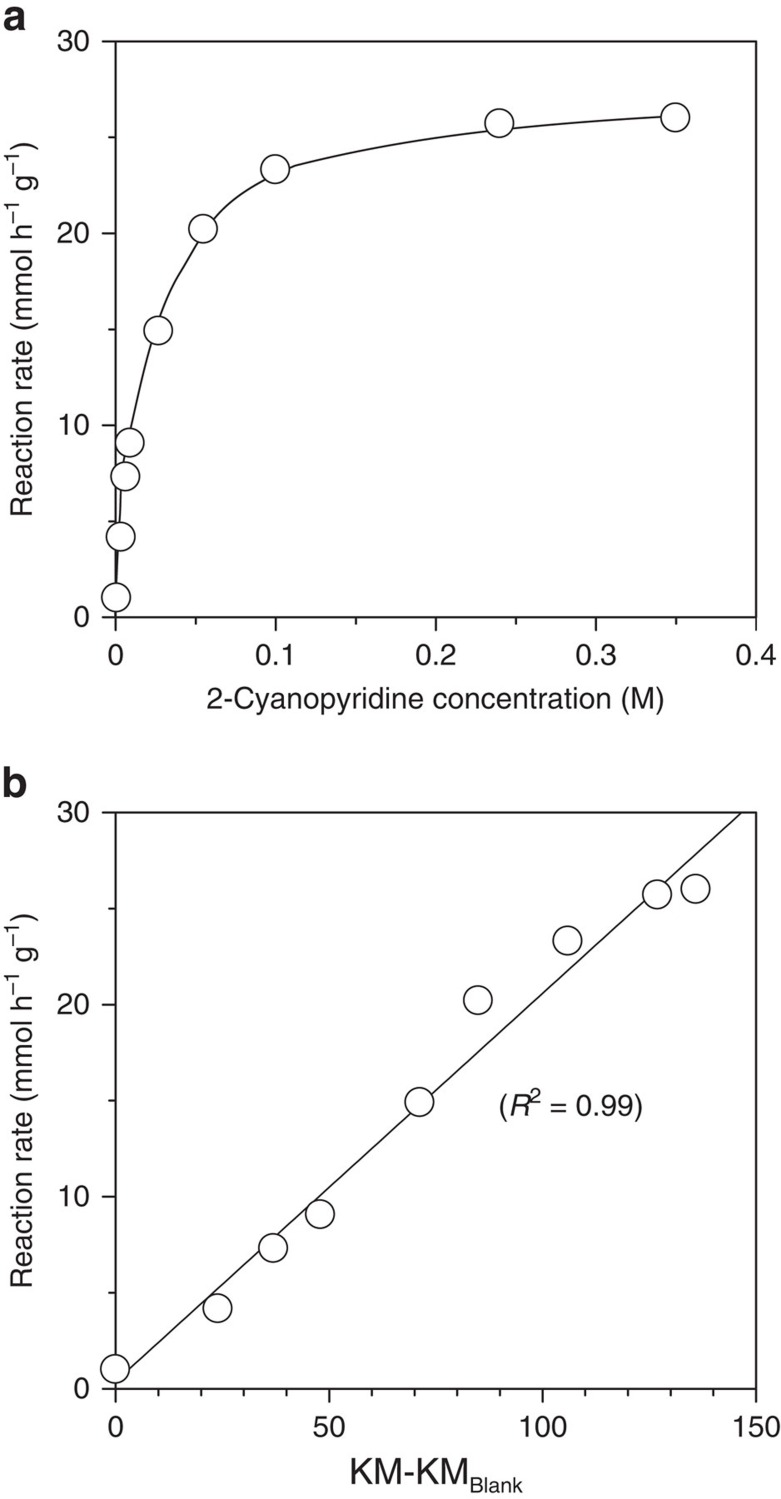
Kinetic studies and correlation between kinetics and UV–vis analyses. (**a**) Reaction rates as a function of 2-cyanopyridine concentration. (**b**) Correlation between the reaction rate and the band intensity at 402 nm in the difference spectra (KM-KM_Blank_) at the same 2-cyanopyridine concentration. KM, Kubelka–Munk.

To ascertain whether the formed CeO_2_-2-cyanopyridine C-T complex truly acts as a catalyst for hydromethoxylation of acrylonitrile, reactions were carried out at various concentrations of 2-cyanopyridine (0–0.34 M), and reaction rates (mmol h^−1^ g^−1^) were determined under conditions where the conversion was below 30%. Figure 4a shows the reaction rate as a function of the concentration of 2-cyanopyridine. The reaction rate increased with increasing 2-cyanopyridine concentration and became asymptotic at high concentrations (≥0.24 M), which was similar in tendency to that of the band intensity in UV–vis spectra against the 2-cyanopyridine concentration (Fig. 3d). The reaction rates were plotted as a function of the band intensity at the same 2-cyanopyridine concentration (Fig. 4b), which provided good correlation between the parameters. The formed complex was concluded to be strongly connected to the active sites. From these results, the uniform CeO_2_-2-cyanopyridine CT complex is most likely formed by the equilibrium adsorption of 2-cyanopyridine on CeO_2_, which serves as the active sites for the reaction.

**Figure 5:**
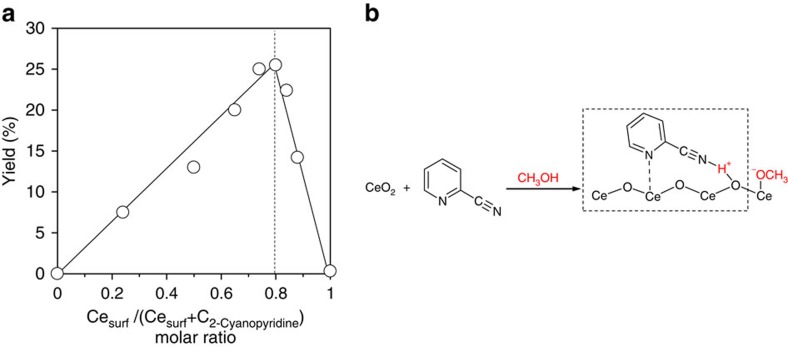
Job's plot and adsorption models of 2-cyanopyridine on CeO_2_. (**a**) Job's plot in complexation of 2-cyanopyridine and CeO_2_. Ce_surf_: surface Ce amount of CeO_2_ (mol), C_2-cyanopyridine_: 2-cyanopyridine amount (mol). Reaction conditions: acrylonitrile (40 mmol), methanol (60 mmol), CeO_2_ (0–10.3 mmol), 2-cyanopyridine (2.0–3.6 mmol), 323 K, air, 0.25 h. Detailed data are described in Supplementary Table 6. (**b**) Adsorption image of 2-cyanopyridine and methanol on CeO_2_.

To determine the surface ratio of 2-cyanopyridine amount (C_2-cyanopyridine_ (mol)) to Ce cation amount on CeO_2_ surface (Ce_surf_ (mol))^37^, the reaction rates were measured by maintaining the total amount of C_2-cyanopyridine_ and Ce_surf_ constant (∼2.0 mmol). Job's plot showed a volcano curve and a maximum yield at the molar ratio of Ce_surf_/(Ce_surf_+C_2-cyanopyridine_)=0.8 (Fig. 5a and Supplementary Table 6), indicating that a 1:4 complex between 2-cyanopyridine and surface Ce cations is preferably formed. Considering that CeO_2_ has only Lewis acid sites as acid sites and Lewis base sites on the surface^42^ and that the basicity of the N atom of pyridine ring is higher than that of the cyano group in 2-cyanopyridine (Supplementary Table 5), the N atom of the pyridine ring in 2-cyanopyridine will be preferentially adsorbed onto the Lewis acid site of the Ce cations of CeO_2_, and the N atom of the cyano group will be close to the O atom of CeO_2_ surface. From the results of the nitrile screening, UV–vis analysis and TG-DTA (Figs 2c and 3, and Supplementary Fig. 5), it is possible that 2-cyanopyridine interacts with CeO_2_ at both the N atoms of the pyridine ring and the cyano group. Further, FTIR analyses demonstrated that the cyano group in 2-cyanopyridine formed a hydrogen bond with the H atom (Supplementary Fig. 7). The precise adsorption structure of the heterogeneous/homogeneous hybrid material is unknown at this point of the research. However, considering that methanol can be dissociated on the acid–base sites on CeO_2_ (ref. 47) and the evidence of the related results presented, such as structural feature of the effective additives (Table 1 and Fig. 2c), UV–vis analyses and TG-DTA of the CeO_2_+2-cyanopyridine (Fig. 3 and Supplementary Fig. 5), and kinetics showing the relationship between the 2-cyanopyridine concentration and reaction rate (Fig. 4), we speculate that an adsorption structure composed of CeO_2_, 2-cyanopyridine and methanol is present, where a N-H^+^-O hydrogen bond exists between the N atom of the cyano group and the O atom of CeO_2_ (Fig. 5b). If such an adsorption structure is formed, expression of strong basicity can be expected due to high stabilization of H^+^ by the neighbouring two heteroatoms (N and O), which may be related to results that effective nitriles with high basicity of the cyano group have high reaction rates (Supplementary Table 5).

### Application to other base-catalysed reactions

To explore the versatility of the catalytic system of CeO_2_+2-cyanopyridine, the catalytic material was applied to transesterification of methyl benzoate with 1-hexanol and Knoevenagel condensation from benzaldehyde and ethyl cyanoacetate, which are known to be catalysed by a base catalyst. The material showed >10-fold higher activity in these reaction systems over that of only CeO_2_ (14-fold for the transesterification and 11-fold for the Knoevenagel condensation, Supplementary Fig. 8). Thus the catalytic system can act as a strong base catalyst, which demonstrates that the catalyst can have widely applied to base-catalysed reactions.

## Discussion

A method for preparing self-assembled heterogeneous/homogeneous hybrid catalysts has been developed. For CeO_2_ and 2-cyanopyridine, by only mixing these materials, the catalytic system has >2,000-fold higher activity for hydromethoxylation of acrylonitrile than when only CeO_2_ or 2-cyanopyridine is used. Formation of the hydrogen bond between the cyano group in 2-cyanopyridine and the H atom derived from methanol was observed. The hydrogen bond between the cyano group in 2-cyanopyridine and the H atom derived from methanol can increase the basicity, which most likely leads to the high activity of the catalytic system. However, the mechanism of the rate enhancement remains unclear because the decisive evidence for the adsorption structure of 2-cyanopyridine, such as whether 2-cyanopyridine is truly adsorbed on the CeO_2_ surface at the N atom in the pyridine ring or whether the hydrogen bond is truly formed in the methanol solvent, could not be determined. Further investigations including DFT calculations of the adsorption state of various nitriles on CeO_2_ and kinetics about nitriles or substrates will be required to clarify the mechanism and the probable adsorption species. Nevertheless, the concept of the self-assembly of metal oxides having acid–base sites and an organic modifier having two functional groups with different basicities or acidities, provides an effective strategy for preparing metal oxide materials that have enhanced catalytic properties.

## Methods

### Materials

All the chemicals for organic reactions were purchased from chemical products corporations and were used without further purification. Acrylonitrile (Tokyo Chemical Industry, >99.0%) 2-cyanopyridine (Tokyo Chemical Industry, >99.0%), methanol (Super dehydrated, Wako Pure Chemical Industries, >99.8%), 3-methoxypropionitrile (Tokyo Chemical Industry, >99.0%), 1,4-dioxane (Wako Pure Chemical Industries, >99.5%), 2-hydroxypyridine (Tokyo Chemical Industry, >98.0%), 2-methylpyridine (Wako Pure Chemical Industries, >98.0%), 2-methoxypyridine (Wako Pure Chemical Industries, >97.0%), 2-acetylpyridine (Wako Pure Chemical Industries, >98.0%), 2-pyridinemethanol (Tokyo Chemical Industry, >98.0%), 2-ethylpyridine (Tokyo Chemical Industry, >98.0%), 2-picolinamide (Tokyo Chemical Industry, >98.0%), 2-(methoxymethyl)-pyridine (Aldrich, >97.0%), 2-pyridineacetonitrile (Tokyo Chemical Industry, >98.0%), pyridine-2-carboxylic acid (Wako Pure Chemical Industries, 98.0%), cyanopyrazine (Tokyo Chemical Industry, >97.0%), 2-cyanopyrimidine (Tokyo Chemical Industry, >98.0%), 2-furonitrile (Wako Pure Chemical Industries, >98.0%), pyridine (Tokyo Chemical Industry, >99.0%), butyronitrile (Wako Pure Chemical Industries, >98.0%), furan (Wako Pure Chemical Industries, >98.0%), benzonitrile (Wako Pure Chemical Industries, >98.0%), 3-cyanopyridine (Wako Pure Chemical Industries, >98.0%), 4-cyanopyridine (Wako Pure Chemical Industries, >98.0%), 2,6-lutidine (Tokyo Chemical Industry, >98.0%), methoxyacetonitrile (Tokyo Chemical Industry, >98.0%), acetone (Wako Pure Chemical Industries, >99.5%), 1-hexanol (Tokyo Chemical Industry, >98.0%), methyl benzoate (Wako Pure Chemical Industries, >98.0%), benzaldehyde (Wako Pure Chemical Industries, >98.0%), ethyl cyanoacetate (Wako Pure Chemical Industries, >98.0%), ethyl α-cyanocinnamate (Tokyo Chemical Industry, >98.0%), ethanol (super dehydrated, Wako Pure Chemical Industries, >99.5%), dodecane (Tokyo Chemical Industry, >99.5%).

### Catalyst

Preparation of CeO_2_ catalyst was carried out by calcining cerium oxide HS (Daiichi Kigenso Kogyo) in 3 h under air at 873 K. The specific surface area (BET method) of pure CeO_2_ was 86 m^2^ g^−1^. Some metal oxides were commercially available or supplied from the Catalysis Society of Japan: ZrO_2_ (Daiichi Kigenso Kogyo, Zr(OH)_2_ was calcined under air at 673 K for 3 h.), MgO (Ube Industries, MgO 500 A, MgO was used after calcining under air at 873 K for 3 h.), TiO_2_ (Nippon Aerosil, P-25), γ-Al_2_O_3_ (Sumitomo Chemical Company, γ-Al_2_O_3_ was used after calcining under air at 873 K for 3 h), Sc_2_O_3_ (Wako Pure Chemical Industries), HfO_2_ (Wako Pure Chemical Industries), Ta_2_O_5_ (Wako Pure Chemical Industries), La_2_O_3_ (Soekawa Chemicals, La(OH)_3_ was calcined under air at 873 K for 3 h), Nb_2_O_5_ (Companhia Brasileira de Metalurgia e Mineracao (CBMM), Nb_2_O_5_·nH_2_O was calcined at 773 K for 3 h). Y_2_O_3_ was prepared by the precipitation method. Y(NO_3_)_3_·nH_2_O (Pure Chemical Industries, >99.9%) was used as a precursor. A precursor (25 g) was dissolved in water (100 ml) and NH_3_aq (1 M) was dropped with stirring. The pH of the solution was set to 10, resulting in a precipitate. The precipitate was filtered and washed with water, following a drying at 383 K overnight (12 h) and calcined under air at 873 K for 3 h. The specific surface area of the metal oxides is summarized in Supplementary Table 7.

### Typical procedure for hydromethoxylation of acrylonitrile

A typical procedure for nucleophilic addition of methanol to acrylonitrile is as follows: CeO_2_ (cerium oxide HS, Daiichi Kigenso Kogyo, calcined at 873 K for 3 h) 172 mg (1 mmol) and methanol 0.64 g (20 mmol) were added to a reaction vessel capped by a rubber plug under air, and the mixture was vigorously stirred at 500 r.p.m. at 323 K under air for 1 h. After the treatment, acrylonitrile 0.53 g (10 mmol) was added into the mixture, and the mixture was constantly stirred during the reaction. The time when acrylonitrile was added in the reactor was defined as zero reaction time. After the reaction, the reaction mixture was filtrated, diluted with acetone, and transferred to a vial. Details of the reaction conditions are described in each result. The products were analysed by gas chromatography (GC) equipped with a FID detector and CP-Sil5 capillary column (length 50 m, i.d. 0.25 mm, film thickness 0.25 μm). Conversion and yield of products were determined based on acrylonitrile by GC using 1,4-dioxane as an internal standard. Products were also identified using standard compounds and GC–MS equipped with the same detector and capillary column. The typical GC chart was shown in Supplementary Fig. 9.

### Typical procedure for transesterification of methyl benzoate with 1-hexanol to hexyl benzoate

CeO_2_ 172 mg (1 mmol), 2-cyanopyridine (1 mmol), methyl benzoate (10 mmol) and 1-hexanol (10 mmol) were added to a reaction vessel, and the mixture was vigorously stirred at 500 r.p.m. at 393 K under air. After the reaction, the reaction mixture was filtrated, diluted with acetone and transferred to a vial. Details of the reaction conditions are described in each result. The products were analysed using GC equipped with a FID detector and CP-Sil5 capillary column (length 50 m, i.d. 0.25 mm, film thickness 0.25 μm). Conversion and yield of products were determined based on methyl benzoate by GC using dodecane as an internal standard. Products were also identified using standard compounds and GC–MS equipped with the same detector and capillary column.

### Typical procedure for Knoevenagel condensation between benzaldehyde and ethyl cyanoacetate to ethyl α-cyanocinnama

CeO_2_ 172 mg (1 mmol), 2-cyanopyridine (1 mmol), ethanol 3.0 g (65 mmol), benzaldehyde (3 mmol) and ethyl cyanoacetate (4 mmol) were added to a reaction vessel, and the mixture was vigorously stirred at 500 r.p.m. at 303 K under air. After the reaction, the reaction mixture was filtrated, diluted with acetone and transferred to a vial. The products were analysed using GC equipped with a FID detector and CP-Sil5 capillary column (length 50 m, i.d. 0.25 mm, film thickness 0.25 μm). Conversion and yield of products were determined based on benzaldehyde by GC using 1,4-dioxane as an internal standard. Products were also identified using standard compounds and GC–MS equipped with the same detector and capillary column.

### Spectroscopic and XRD analyses

UV–vis diffuse reflectance spectra were measured with Shimadzu UV 2450 spectrophotometer with the integration sphere diffuse reflectance attachment (ISR-2200 Shimadzu) and a photomultiplier detector. Pure BaSO_4_ was used as a reference sample. The solution samples were added in a transparent quartz cell and the cell was located at the integration sphere diffuse reflectance attachment. The spectra were measured in the region of 350–800 nm at room temperature. The scan speed is middle and the resolution of spectra is 0.1 nm. The sample without CeO_2_ sample was measured by the conventional transmission method on the same spectrophotometer using the 1-cm path length transparent quartz cell.

XRD patterns were recorded by Rigaku MiniFlex600 with Cu *K*α (40 kV, 15 mA) radiation and D/tex Ultra2 detector. The diffractometer data were recorded for 2*θ* values between 20 and 70° at a scanning rate of 10° min^−1^ at a resolution of 0.02°.

FTIR spectra were recorded with a NICOLET 6700 spectrometer (Thermo Scientific) equipped with a liquid nitrogen-cooled MCT (HgCdTe) detector (resolution 4 cm^−1^), using an *in situ* IR cell with CaF_2_ windows, which was connected to a conventional gas flow system. CeO_2_ sample (∼80 mg) was pressed into a self-supporting wafer (20 mm diameter) and mounted into the IR cell. Adsorption of methanol and 2-cyanopyridine was carried out in the following method: The catalyst was preheated at 873 K under He(50 ml min^−1^)/O_2_(10 ml min^−1^) flow for 10 min. Then the catalyst was cooled down to 323 K under a He flow. Methanol (4 μl) was injected into the gas line heated at 523 K under a He flow, which was fed to the *in situ* IR cell. After the spectrum was stable, 2-cyanopyridine was introduced under He into the IR cell, and the spectrum change was monitored. Spectra were obtained by subtraction of the reference spectrum of CeO_2_ measured at 323 K under He flow.

## Additional information

**How to cite this article:** Tamura, M. *et al*. Self-assembled hybrid metal oxide base catalysts prepared by simply mixing with organic modifiers. *Nat. Commun.* 6:8580 doi: 10.1038/ncomms9580 (2015).

## Supplementary Material

Supplementary Information Supplementary Figures 1-9, Supplementary Tables 1-7 and Supplementary References

## Figures and Tables

**Figure 1:**
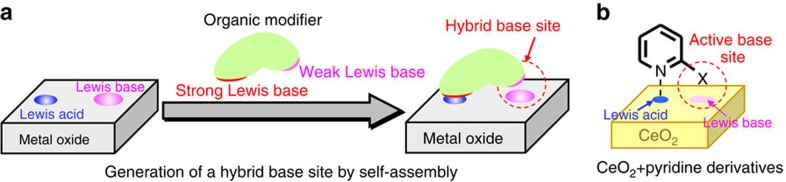
Schematic images of strategy for design of organic compound-modified metal oxides. (**a**) Self-assembled heterogeneous/homogeneous hybrid catalyst composed of a metal oxide and an organic modifier. (**b**) Self-assembled heterogeneous/homogeneous hybrid catalyst based on CeO_2_ and pyridine derivatives.

**Table 1:**
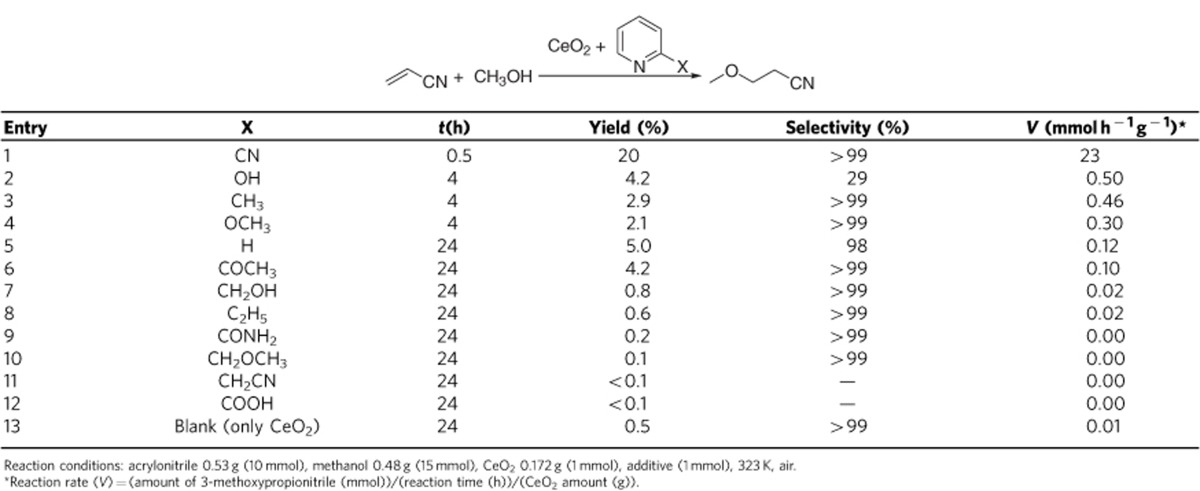
Comparison of catalysts composed of CeO_2_ and pyridine derivatives in hydromethoxylation of acrylonitrile.
