# Supplementary material for: Self-assembled hybrid metal oxide base catalysts prepared by simply mixing with organic modifiers
Source: Nat Commun. 2015 Oct 5;6:8580. doi: 10.1038/ncomms9580 (PMC4600743; doi:10.1038/ncomms9580)
Supplement: Supplementary Information — Supplementary Figures 1-9, Supplementary Tables 1-7 and Supplementary References [file ncomms9580-s1.pdf]

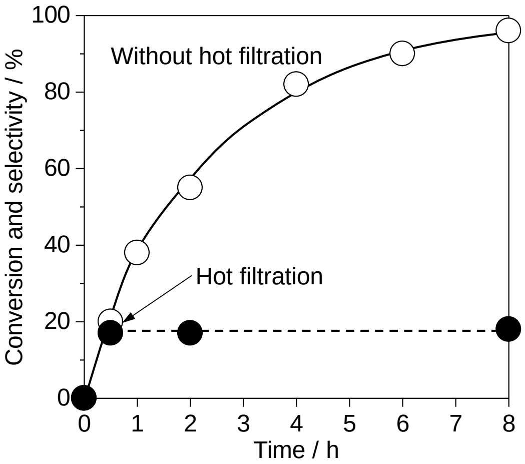

Supplementary Figure 1 Leaching test for hydromethoxylation of acrylonitrile with methanol over CeO_2 and 2-cyanopyridine. (○) without hot filtration, (●) Hot filtration.

Reaction conditions: acrylonitrile (10 mmol), methanol (15 mmol), CeO_2 (1 mmol), 2-cyanopyridine (1 mmol), 323 K, air.

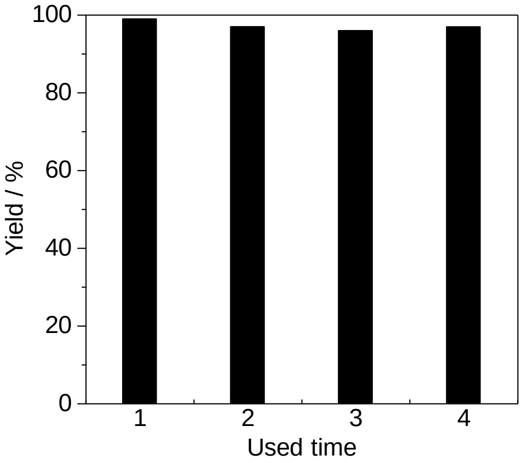

Supplementary Figure 2 Reusability of CeO_2 in hydromethoxylation of acrylonitrile over CeO_2 and 2-cyanopyridine.

Reaction conditions: acrylonitrile (10 mmol), methanol (15 mmol), CeO_2 (1 mmol), 2-cyanopyridine (1 mmol), 323 K, 10 h, air.

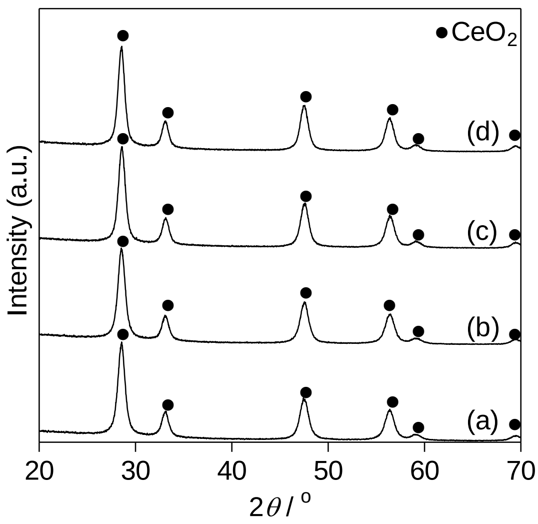

Supplementary Figure 3 XRD patterns of CeO_2 samples.

(a) fresh, (b) after the first reaction, (c) after the second reaction, (d) after the third reaction

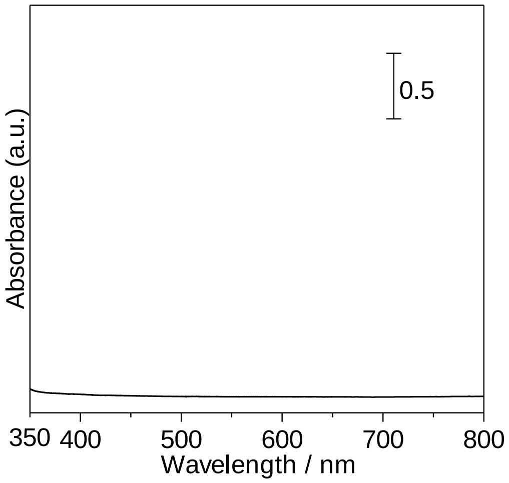

Supplementary Figure 4 UV-vis spectrum of 2-cyanopyridine in methanol.

Conditions: 2-cyanopyridine (2 mmol), methanol (10 mmol).

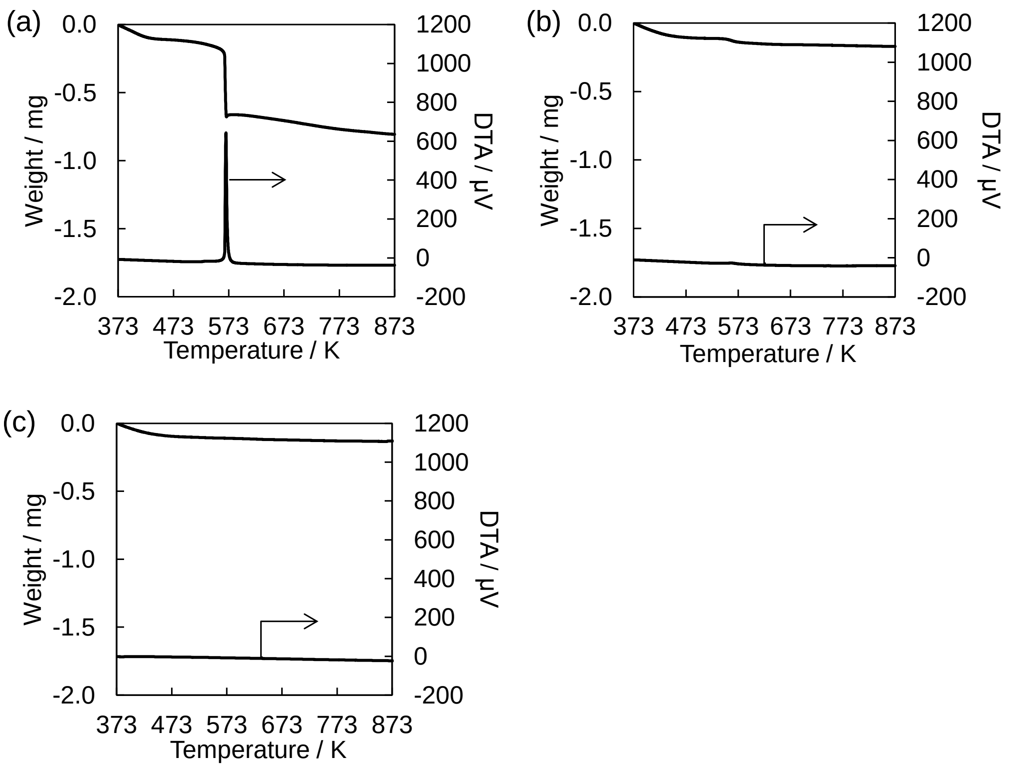

Supplementary Figure 5 TG-DTA profiles of (a) CeO_2 after the reaction, (b) CeO_2 washed with methanol (100 ml) after the reaction and (c) fresh CeO_2.

Reaction conditions: acrylonitrile (10 mmol), methanol (15 mmol), CeO_2 (1 mmol), 2-cyanopyridine (1 mmol), 323 K, air, 0.5 h. Measurement conditions: sample weight (10 mg), heating rate (10 K min^{-1}), under air.

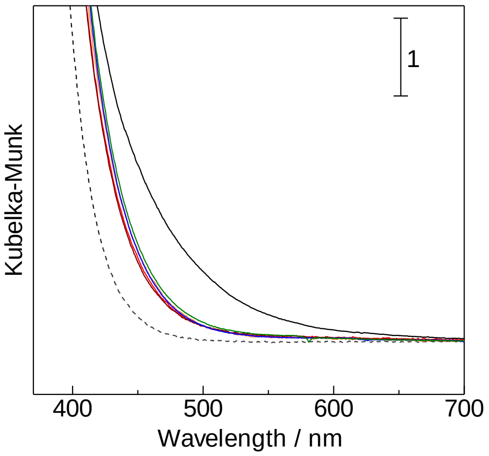

Supplementary Figure 6 UV-vis spectra of the reaction mixtures with various ratios of methanol to 2-cyanopyridine.

Dotted line: blank, solid line: 2-cyanopyridine only, red line: 2-cyanopyridine/methanol=0.067, orange line: 2-cyanopyridine/methanol=0.1, brown line: 2-cyanopyridine/methanol=0.2, blue line: 2-cyanopyridine/methanol=1, green line: 2-cyanopyridine/methanol=2.

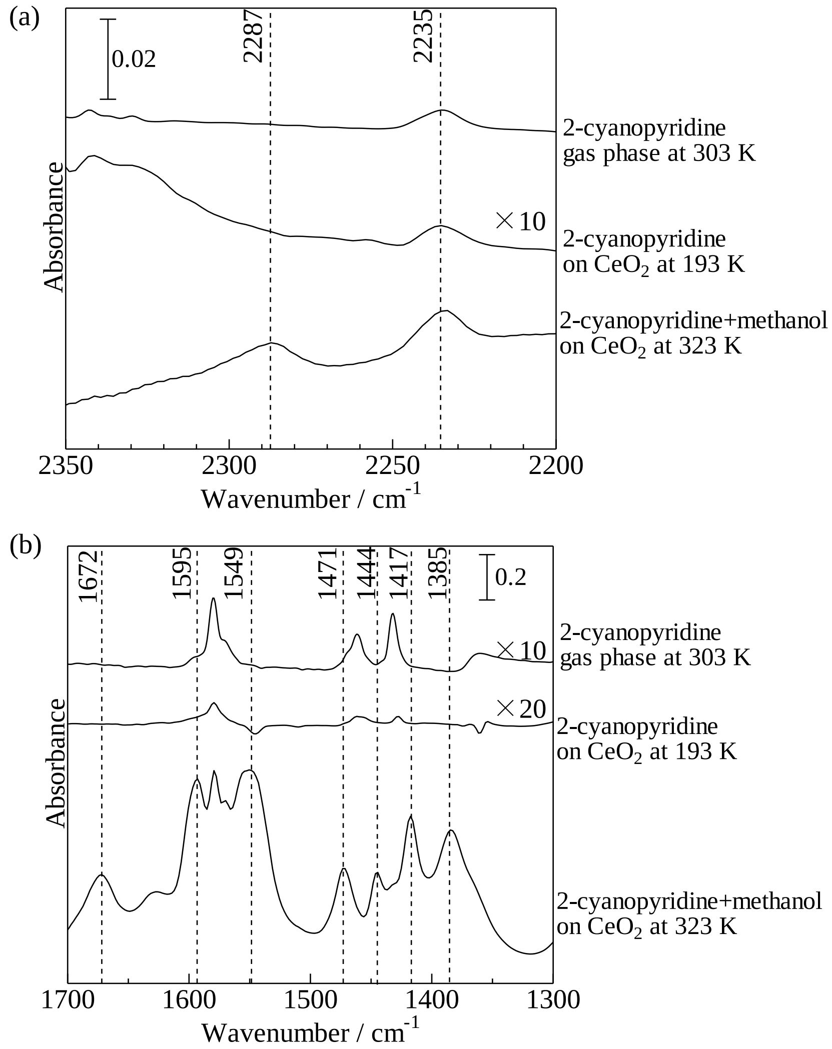

Supplementary Figure 7 FTIR spectra of 2-cyanopyridine and/or methanol adspecies on CeO_2.
(a) 2200-2350 cm^{-1}, (b) 1300-1700 cm^{-1}.

At the region between 1300 and 1700 cm^{-1}, most of 2-cyanopyridine reacted to provide the amide and amide anion species on the CeO_2 surface, which were observed at 1385, 1417, 1444, 1471, 1549, 1595 and 1672 cm^{-1} (Supplementary Figure 7(b)). On the other hand, at the region between 2200 and 2350 cm^{-1} (Supplementary Figure 7(a)), two ν(CN) bands at 2235 and 2287 cm^{-1} were observed. The band at 2235 cm^{-1} is in good agreement with the band position of gaseous 2-cyanopyridine or adsorbed 2-cyanopyridine on CeO_2. Taking into consideration that about 10 cm^{-1} band shift of ν(CN) on CeO_2 was observed in the case of CD_3CN in the our previous work[1], the CN group in 2-cyanopyridine observed at 2235 cm^{-1} will not interact with the CeO_2 surface. On the other hand, the band at 2287 cm^{-1} was shifted by 43 cm^{-1} from 2235 cm^{-1}. In general, the formation of H-bonding between surface OH species and CN group provides 0-40 cm^{-1} shift of ν(CN)[2,3] Therefore, the band at 2287 cm^{-1}can be assigned to ν(CN) with a hydrogen bond.

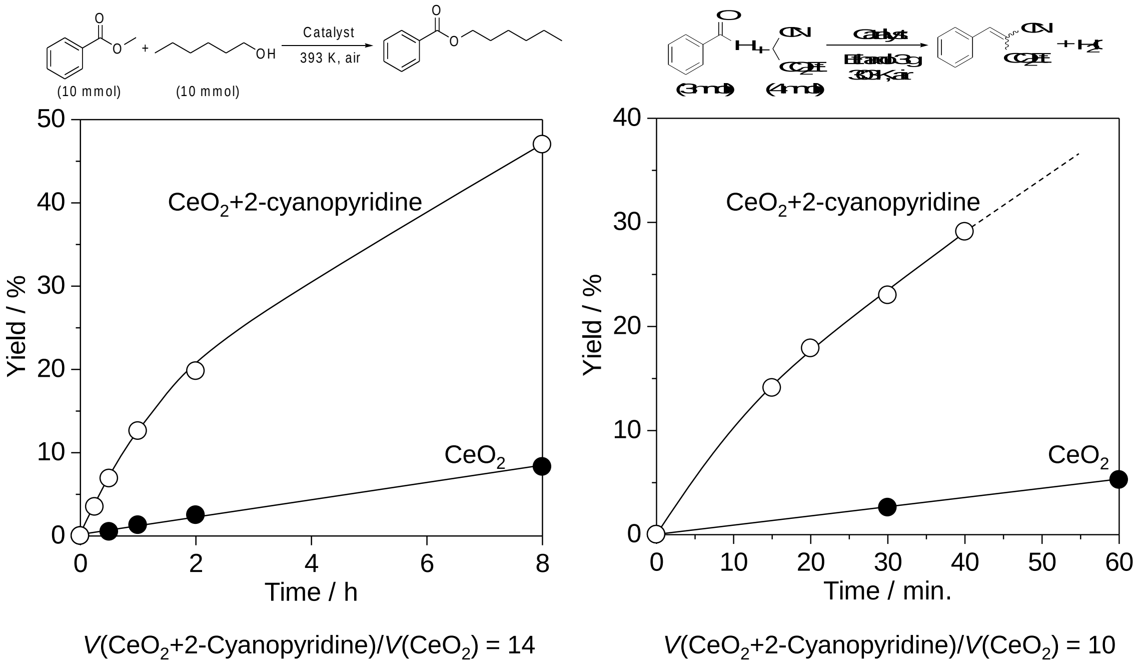

Supplementary Figure 8 Comparison of activities between CeO_2+2-cyanopyridine and only CeO_2 in the transesterification reaction and Knoevenagel condensation.

V(CeO_2) and *V*(CeO_2+2-cyanopyridine) are the initial reaction rates over CeO_2 and CeO_2+2-cyanopyridine system, respectively.

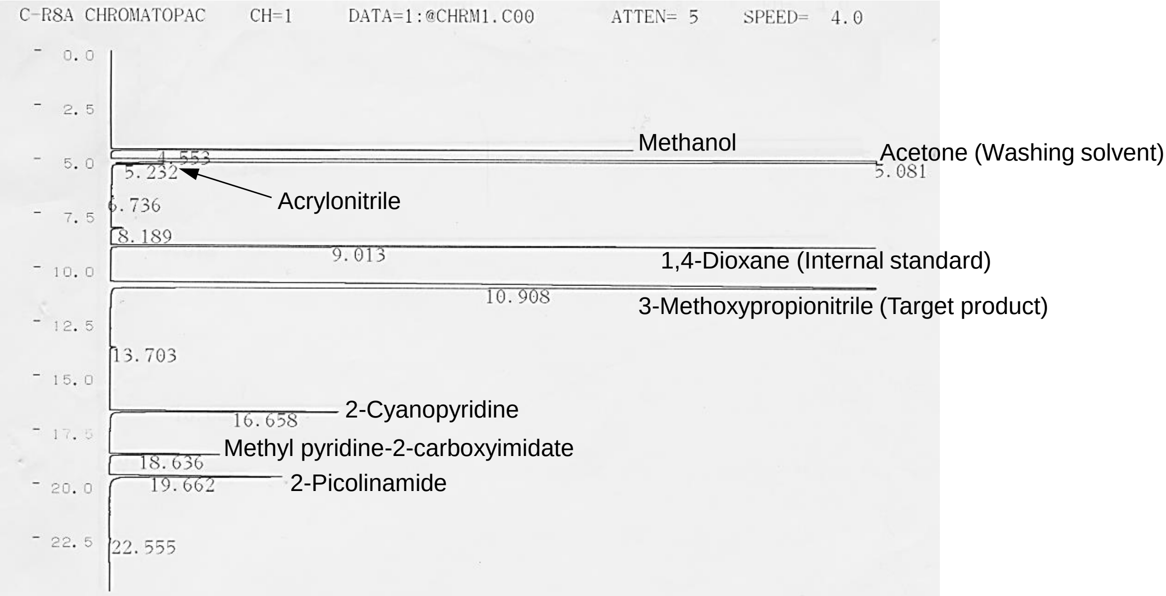

C-R8A CHROMATOPAC CH=1 Report No.=1549 DATA=1:@CHRM1.C00 13/05/29 17:47:28

** CALCULATION REPORT **

CH	PKNO	TIME	AREA	HEIGHT	MK	IDNO	CONC	NAME
1	1	4.553	43459	21871			1.2286	
	2	5.081	2952586	798013	S		83.4696	
	3	5.232	3606	2000	T		0.1019	
	4	6.736	230	96			0.0065	
	5	8.189	1801	558			0.0509	
	6	9.013	84477	32156			2.3882	
	7	10.908	376621	37116			10.6471	
	8	13.703	824	256			0.0233	
	9	16.658	31464	9566			0.8895	
	10	18.636	11813	4599			0.3339	
	11	19.662	30107	7213			0.8511	
	12	22.555	331	65			0.0094	
	TOTAL		3537316	913508			100	

Supplementary Figure 9 GC chart of the reaction mixture for hydromethoxylation of acrylonitrile over CeO_2 and 2-cyanopyridine after 8 h.

Reaction conditions: acrylonitrile (10 mmol), methanol (15 mmol), CeO_2 (1 mmol), 2-cyanopyridine (1 mmol), 323 K, air.

Supplementary Table 1 Hydromethoxylation of acrylonitrile by various metal oxides.

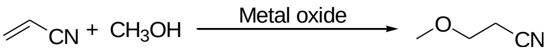

Metal oxide	*t* / h	Yield / %	Selectivity / %	*V* / mmol h^{-1} g_{cat}^{-1}
CeO_2	48	1.1	>99	0.01
La_2O_3	24	9.0	99	0.22
MgO	24	3.8	99	0.09
Y_2O_3	12	1.2	93	0.06
Sc_2O_3	24	1.8	99	0.04
Eu_2O_3	48	3.4	98	0.04
ZrO_2	48	0.5	>99	0.01
Al_2O_3	48	0.7	>99	0.01
TiO_2	48	<0.1	-	0.00
Nb_2O_5	48	<0.1	-	0.00
HfO_2	48	<0.1	-	0.00
Ta_2O_5	48	<0.1	-	0.00
Blank	48	<0.1	-	-

Reaction conditions: acrylonitrile (10 mmol), methanol (20 mmol), metal oxide (172 mg), 323 K, air.

Supplementary Table 2 Hydromethoxylation of acrylonitrile by various metal oxides and 2-cyanopyridine.

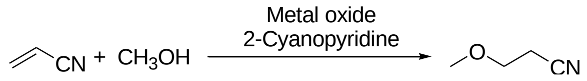

Metal oxide	t / h	Yield / %	Selectivity /%	V / mmol h^{-1} g_{cat}^{-1}
CeO_2	0.5	18	>99	21
La_2O_3	24	18	99	0.44
MgO	24	6.1	99	0.15
Y_2O_3	12	1.6	96	0.08
Sc_2O_3	24	1.6	99	0.04
Eu_2O_3	48	1.8	99	0.02
ZrO_2	48	0.8	>99	0.01
Al_2O_3	48	1.2	>99	0.01
TiO_2	48	0.1	>99	0.00
Nb_2O_5	48	<0.1	-	0.00
HfO_2	48	<0.1	-	0.00
Ta_2O_5	48	<0.1	-	0.00
Blank	48	<0.1	-	-

Reaction conditions: acrylonitrile (10 mmol), methanol (20 mmol), metal oxide (172 mg), 2-cyanopyridine (2 mmol), 323 K, air.

Supplementary Table 3 Time-course of hydromethoxylation of acrylonitrile over CeO_2 and 2-cyanopyridine.

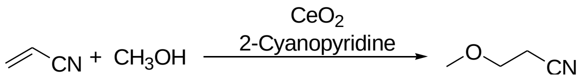

t / h	Conv. / %	Yield / %	Selectivity / %	Amount of products derived from 2-cyanopyridine / mmol	
				Methyl pyridine-2-carboxyimidate	2-Picolinamide
0	0	0	-	0.02	0.42
0.5	20	20	>99	0.03	0.42
1	38	38	>99	0.04	0.42
2	55	55	>99	0.05	0.43
4	82	81	99	0.08	0.44
6	90	90	>99	0.09	0.46
8	96	95	99	0.10	0.46
10	98	98	>99	0.11	0.46

Reaction conditions: acrylonitrile (10 mmol), methanol (15 mmol), CeO_2 (1 mmol), 2-cyanopyridine (1 mmol), 323 K, air.

Supplementary Table 4 Comparison of the initial reaction rates for hydromethoxylation over CeO_2 and various additives.

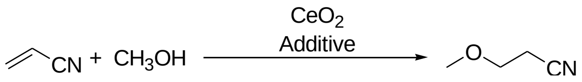

Entry	Additive		t / h	Conv. / %	Yield / %	V / mmol h^{-1} g^{-1}
1	2-Cyanopyridine	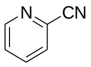	0.5	20	20	23
2	Cyanopyrazine	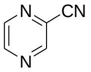	1	18	18	10
3	2-Cyanopyrimidine	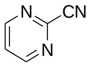	1	11	11	6.3
4	2-Furonitrile	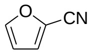	1	9.4	9.3	5.4
5	Methoxyacetonitrile	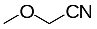	3	21	21	4.1
6	Pyridine	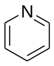	24	5.0	5.0	0.12
7	Butyronitrile	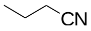	24	4.6	4.5	0.11
8	Furan	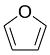	48	2.4	2.3	0.03
9	Benzonitrile	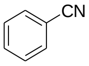	48	1.0	1.0	0.01
10	3-Cyanopyridine	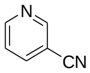	48	0.87	0.87	0.01
11	4-Cyanopyridine	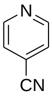	48	0.61	0.61	0.01
12	None		48	1.1	1.1	0.01

Reaction conditions: acrylonitrile (10 mmol), methanol (20 mmol), CeO_2 (1 mmol), additive (2 mmol), 323 K, air.

Supplementary Table 5 Acidities of various additives calculated by DFT (B3LYP/6-311++G(d,p)).

Additive	ΔE of protonation (kJ/mol)	pK_a of conjugate acid[a]	V / mmol h^{-1} g^{-1}
2-Cyanopyridine	-907 (1N)<br>-834 (CN)	-0.26	23
Cyanopyrazine	-857 (4N)<br>-852 (1N)<br>-807 (CN)		10
5-Fluoro-2-cyanopyridine	-878 (1N)<br>-825 (CN)		8.4
2-Cyanopyrimidine	-864 (1N)<br>-819 (CN)		6.3
2-Furonitrile	-833 (CN)<br>-662 (O)		5.4
Methoxyacetonitrile	-803 (CN)<br>-758 (O)		4.1
Pyridine	-966	5.67	0.12
Butyronitrile	-830		0.11
Furan	-719		0.03
Benzonitrile	-850		0.01
3-Cyanopyridine	-909 (1N)<br>-826 (CN)		0.01
4-Cyanopyridine	-915 (1N)<br>-810 (CN)	1.90	0.01

[a] "Kagaku-Binran Kiso-Hen II (Chemistry Handbook Basic Part II)", 5th edition, edited by Chemical Society of Japan, Maruzen, Tokyo, 2004.

Supplementary Table 6 Detailed data for the job's plot of the reaction rates as a function of $Ce_{surf}/(Ce_{surf}+C_{2\text{-cyanopyridine}})$ molar ratio.

Entry	Ce amount /mmol	Ce_{surf} /mmol	$C_{2\text{-cyanopyridine}}$ /mmol	$Ce_{surf}/(Ce_{surf}+C_{2\text{-cyanopyridine}})$	Yield /%
1	10.26	2.00	0.00	1.00	0.31
2	10.00	1.95	0.25	0.88	14.2
3	9.23	1.80	0.35	0.84	22.4
4	8.72	1.70	0.43	0.80	25.5
5	8.21	1.60	0.55	0.74	25
6	7.69	1.50	0.79	0.65	20
7	5.13	1.00	1.00	0.50	13
8	2.56	0.50	1.50	0.24	7.5
9	0.00	0.00	2.01	0.00	0

Reaction conditions: acrylonitrile (40 mmol), methanol (60 mmol), 323 K, 0.25 h, air. Ce_{surf}: surface Ce amount of CeO_2 (mol), $C_{2\text{-cyanopyridine}}$: 2-cyanopyridine amount.

Supplementary Table 7 Specific surface area of metal oxides.

Metal oxide	S_{BET} / $m^2 g^{-1}$
CeO_2	86
La_2O_3	7.0
MgO	20
Y_2O_3	62
Sc_2O_3	5.0
Eu_2O_3	5.7
ZrO_2	91
Al_2O_3	182
TiO_2	48
Nb_2O_5	46
HfO_2	11
Ta_2O_5	3.2
Blank	-

Supplementary References

1. Tamura, M., Shimizu, K.-i. & Satsuma, A. Comprehensive IR study on acid/base properties of metal oxides, *Appl. Catal. A*, **433-434,** 135-145 (2012).
2. Morterra, C., Cerrato, G., Novarino, E. & Peñarroya Mentruit, M. On the Adsorption of Acetonitrile on Pure and Sulfated Tetragonal Zirconia (t-ZrO_2), *Langmuir*, **19**, 5708-5721 (2003).
3. Tamura, M., Shimizu, K.-i. & Satsuma, A. Comprehensive IR study on acid/base properties of metal oxides, *Appl. Catal. A*, **433-434,** 135-145 (2012).
